# Attitudes towards prisoners, as reported by prison inmates, prison employees and college students

**DOI:** 10.1186/1471-2458-7-71

**Published:** 2007-05-04

**Authors:** Ellen Kjelsberg, Tom Hilding Skoglund, Aase-Bente Rustad

**Affiliations:** 1 Centre for Research and Education in Forensic Psychiatry, Psychiatric Division, Ullevaal University Hospital, KPS, Building 7 Gaustad, N-0320 Oslo, Norway; 2 Psychiatric High Security Unit, Psychiatric Division, Ullevaal University Hospital, Oslo, Norway

## Abstract

**Background:**

Positive attitudes towards prisoners are important in securing the effectiveness of various correctional rehabilitation programs and the successful reintegration of prisoners after release. We wanted to investigate the attitudes towards prisoners among prison inmates, prison employees and college students.

**Methods:**

The Attitudes Toward Prisoners scale was completed by 298 inmates in 4 Norwegian prisons, 387 employees working in the same prisons, and 183 college students. In addition, all respondents were asked a number of general questions about prisoners, crime and punishment.

**Results:**

The study groups differed significantly in their attitudes towards prisoners, as measured by the Attitudes Toward Prisoners scale, with prison inmates holding the most positive attitudes. Prison officers held more negative attitudes than other prison employees. Prison employees working in female-only facilities held more positive attitudes than those working in male-only facilities. Students differed significantly in their attitudes, with those studying business economics holding more negative attitudes than those studying nursing. A number of strong correlations emerged between negative attitudes towards prisoners and more pessimistic and punitive answers on general questions about prisoners, crime and punishment.

**Conclusion:**

The attitudes towards prisoners differed markedly among the groups investigated. The findings could have important implications, particularly for the preventive work carried out in our prisons. Whether attitudes toward prisoners can be influenced by educational programs and the dispersion of factual information needs to be investigated.

## Background

One of the most important goals of any correctional facility is to help the prisoners change their criminal behaviour and avoid re-offending after release. Consequently, our correctional facilities offer a number of rehabilitation programs [1]. It is important that the professionals providing these programs hold positive attitudes towards the prisoners. In this context, positive attitudes signify a view of prisoners as normal persons capable of positive change, whereas negative attitudes signify a view of prisoners as incurably deviant individuals [2].

Prison officers' attitudes towards prison inmates are important. Positive attitudes held by prison officers have been shown to be critical in facilitating change prior to successful release from prison [3]. Prison officers are in day-to-day interaction with the inmates and in this unique situation they have the power to enhance or undermine the primary goals of the correctional institution where they work. The prison officers' work situation has been described as psychosocially taxing [4]. Negative attitudes have been demonstrated in some studies [5]. Prison officers have even been described as cynical, authoritarian and pessimistic [6]. Some seem to hold the view that the correctional facilities' main objective is to offer passive storage of criminals rather than to promote rehabilitation and prevention [7]. Such negative attitudes seem to be more common in correctional facilities with little focus on rehabilitation than in institutions with a strong focus on such goals [7].

In addition, the inmates' attitudes towards their fellow prisoners, and indeed the inmates' attitudes toward themselves, i.e. their self-esteem, are important, because these attitudes are likely to influence the way prisoners respond to the correctional regime and the over-all effectiveness of the various rehabilitation programs offered [2]. Whether rehabilitation will be successful will also depend upon the attitudes held by the population into which the prisoner will be released.

We have found some studies on the attitudes towards prisoners in different populations [2,8-17]. However, the study samples have often been small and not always fully representative of the populations they have intended to investigate. Particularly prisoners' attitudes have been sparsely studied. We found only one study [2] citing such results. In addition, knowledge regarding the influence of age, gender and other demographic factors on these attitudes in different populations is lacking.

We wanted to increase the knowledge in the field by conducting the study presented here. The aim of the study was threefold:

1. – to investigate the attitudes towards prisoners in a large population of prison inmates, both males and females, and compare them with the attitudes held by the prison employees in the same correctional facilities and the attitudes held by young college students. We chose the Attitudes Toward Prisoners scale (ATP), developed by Melvin, Gramling and Gardner [2].

According to basic theories in social psychology [18,19] knowledge and beliefs play a central role in the formation or change in attitudes. Accordingly, we wanted:

2. – to explore the link between attitudes towards prisoners, as measured by ATP, and more general beliefs about prisoners, crime and punishment. In order to be able to do this, we developed a questionnaire intended to gauge the respondents' beliefs and off-the-cuff knowledge about such issues.

As we collected a large data base we also wanted:

3. – to investigate some of the psychometric properties of the ATP scale, particularly the underlying factor structure.

In accordance with previous studies we expected prison inmates to hold the most positive attitudes towards prisoners, as measured by the ATP. We expected students' attitudes to be most negative and prison employees to hold an intermediate position. In addition, we expected the responses to our more general questions about crime and criminality to be influenced by the respondents' general attitudes towards prisoners and consequently correlate with the ATP. As previous research literature had reported good psychometric properties of the ATP, we expected findings of the same nature.

## Methods

### The Attitudes Toward Prisoners scale (ATP)

We chose the ATP because it is often encountered in the available research literature [2,10,12,15] and it has good psychometric properties [2]. In the original study the ATP had a split-half reliability fluctuating between .84 and .92 in five different samples. Test-retest reliability was .82. Validity was good and there was no evidence of response distortion.

The ATP scale [20] contains 36 statements about attitudes towards prisoners, like "Most prisoners can be rehabilitated" and "Prisoners respect only brute force", and to which participants are asked to rate their degree of agreement. The ATP uses a Likert type scale with responses ranging from Disagree strongly, Disagree and Undecided, to Agree, and Agree strongly. After reversal of 19 of the 36 items, each item receives a score from 1 to 5, with 1 representing the most negative attitude and 5 the most positive attitude. A constant of 36 is removed from the sum score, making the possible scoring range 0 to 144. Positive scores suggest that prisoners are viewed as normal persons capable of positive change, whereas negative scores reflect the view that prisoners are basically deviant individuals incapable of positive change.

The instrument was translated to Norwegian by us and back-translated to English by a professional translator with satisfactory results. Participants could choose between the Norwegian and the English version of the ATP.

### The Questionnaire

The authors developed a set of questions intended to gauge the respondents' off-the-cuff beliefs about prisoners, crime and punishment. In the instruction, we stressed that we wanted the respondents' intuitive estimates rather than factual knowledge: no answers would be classified as correct or incorrect. The questions from the questionnaire are presented in Tables 3 and 4, with questions asking for percentage estimates in Table 3 and questions with categorical answers in Table 4.

### Research ethics

The study was approved by the Regional Committee for Medical Research Ethics, Eastern Norway (Ref. 530-04210). Verbal consent was obtained from all participants. No incentives were given for participation.

### The prison inmates

Two of Norway's largest male-only prisons, Oslo and Ullersmo, and the country's two female prisons, Bredtveit and Fredrikstad, were asked to, and agreed to, participate in the present study.

At the time of the study, these 4 prisons accommodated 580 prisoners, 504 males and 76 females. A total of 305 prison inmates (53%), 254 (50%) of the males and 51 (67%) of the females, agreed to participate in the study. In one of the prisons there were obvious shortcomings in the routines for asking prisoners to participate in the study, and the later collection of completed questionnaires. These practical difficulties occurred in a random fashion throughout the prison. As a result, the attrition rate at that particular site was higher (68%) than in the 3 remaining sites, where attrition was 44, 40 and 8%, respectively.

The mean age of the participating prisoners was 34 years (SD = 9.4, range 18–72). This is close to the mean age of the total prison population, 34.8 years, at the time of the investigation (Norwegian Correctional Services' internal statistics). Among the participating inmates, 26% were remand prisoners and 74% were serving sentences. The mean number of prison sentences served before the current one was 4.2 (SD = 7.2, median = 2, range 0–52). A total of 16% of the inmates chose the English version of the questionnaire, 32% of the remanded and 10% of the sentenced prisoners.

### The prison employees

Altogether 648 persons were employed in the 4 participating prisons at the time of the study. Of these, a number of persons were unavailable for a variety of reasons, such as sick-leave, leave-of-absence, and work assignments outside the prison. In addition, a number of employees stated that they would have participated if they had had the time. In the end, a total of 390 employees participated in the study, resulting in a participation rate of 60%.

Among the 390 participating prison employees there were 56% males and 44% females; the mean age was 41.3 years (SD = 11.0, range 20–69). On average they had worked for 11.1 years (SD = 8.9, range 0.1–42) in the correctional system. A total of 59% worked as prison officers; 96% of these had graduated from the Correctional Service of Norway Staff Academy. Among the remaining employees, 22% occupied purely administrative positions and 78% were engaged in a variety of other positions, such as occupational therapist, teacher, librarian, psychologist and kitchen and maintenance worker. To secure anonymity the respondents were not asked to specify their profession.

### The college student sample

Business management students and history students from Agder College and nursing students from Buskerud College were asked to participate. The questionnaires were distributed to all students present at the end of arbitrarily chosen lectures. Those who did not want to participate were asked to simply return a blank form.

Among the 198 students approached, 14 students chose not to participate. Hence, the student sample included 184 respondents, 93% of those approached: 83 studying business management, 81 studying nursing, and 20 history students. The mean age was 25.3 years (SD = 7.7, median 22, range 19–52). One third of the total student sample consisted of males, with a surplus of male students in business management (53%) and of female students in nursing (89%) and history (55%).

### Handling of missing data

The ATP and the questionnaire were on reverse sides of the same sheet of paper. Some of the respondents had obviously not turned the page. Hence, 8 had answered the questionnaire, but had missed the ATP on the reverse side, while 33 had completed the ATP, but had failed to answer the questionnaire. Accordingly, not all results add up to the total number of respondents, 879.

In addition to the 8 forms with all ATP items missing, 3 forms with more than 4 items missing were excluded from the ensuing ATP analyses. Forms with less than 4 items missing were retained, substituting missing scorings with a score of 3 ("uncertain") in order to be able to compute an ATP sum score. No specific ATP item had a superabundance of missing scores. Hence, the study sample includes 868 satisfactorily completed ATP forms.

Missing scores were more frequently encountered in the questionnaire. As already stated, 33 had not completed the reverse side of the ATP. In addition, some had left a number of individual items open. Although the questionnaire asked for opinions, not facts, some questions might have been perceived as "difficult" to answer. Among the 846 who had completed the questionnaire the number of missing answers on individual items varied between zero and 5%, with prison inmates having more missing answers. In addition, the responses from 4 prison inmates on questions about percentages had to be discarded, as they obviously had no knowledge about the concept.

### Statistical procedures

Differences in the ATP scorings in the various sub-groups were explored using Student's t-test or One-way ANOVA with Scheffe's post-hoc test, depending on the number of sub-groups being compared. To investigate whether a number of demographic factors were independent and significant contributors to the differences found within each group, we first established Pearson's correlations between the ATP scores and these demographic factors. Next, all factors thus found to be significantly correlated with ATP were subjected to multivariate statistics in the form of stepwise (forward conditional) linear regression analyses.

Group differences found in the answers to the questionnaire were explored using One-way ANOVA with Scheffe's post-hoc test on continuous variables and Pearson's Chi square test on categorical variables. In addition, Pearson's correlations between the ATP and all questionnaire responses were calculated.

Lastly, we used our data set to subject the 36 items of the ATP to principal components analysis (PCA), in order to test the intended uni-dimensional factor structure of the scale. Cronbach's alpha was also calculated.

The Statistical Package for the Social Sciences, SPSS [21], was used throughout.

## Results

### The ATP

All main results are presented in the tables. Table 1 presents the mean ATP scores in the different samples and sub-samples, with t-test and one-way ANOVA statistics, highlighting statistically significant group differences. While employees in female-only prisons held more positive attitudes than employees in male-only prisons, in both prison categories prison officers held more negative attitudes than the rest of the employees. Thus, the relative ranking between the two sub-groups of employees were retained, irrespective of the gender of the prisoners.

**Table 1:** Attitudes Toward Prisoners scale (ATP) mean scores in different sub-samples.

**Study sample**	**N**	**Mean ATP **(SD, range)	**Student's t-test **(p-value)
**Total sample**			
Total	868	97 (17.9, 33–140)	
			
Males	511	98 (18.5, 33–140)	
Females	335	97 (17.0, 48–137)	0.76 (.45)
			
Prison inmates	298	106 (16.5, 50–140)	
Prison employees	387	93 (16.6, 34–134)	
College students	183	91 (17.6, 33–130)	60.3 (<.001)^1^

**Prison inmates**			
Males	247	105 (16.3, 67–140)	
Females	51	107 (17.7, 50–137)	-0.65 (.52)
			
Remanded	70	102 (16.3, 67–135)	
Convicted	203	107 (16.8, 50–140)	-2.01 (.045)

**Prison employees**			
Males	204	92 (16.2, 34–134)	
Females	163	96 (16.8, 48–129)	-2.50 (.01)
			
Prison officers	222	90 (16.8, 34–129)	
Other employees	151	98 (15.6, 62–134)	-4.87 (<.001)
			
Working in male prison	321	92 (17.0, 34–134)	
Working in female prison	66	100 (12.2, 68–127)	-3.81 (<.001)

**College students**			
Males	60	86 (20.9, 33–127)	
Females	121	93 (15.2, 61–130)	-2.53 (.01)
			
Business economics	82	85 (17.9, 33–130)	
History	20	95 (19.4, 44–125)	
Nursing	81	96 (15.2, 62–127)	8.46 (<.001) ^2^

As not all informants have answered all demographic questions, sub-sample numbers do not always add up to the sum total.

^1 ^One-way ANOVA. Scheffe's post-hoc tests: Inmates statistically different from prison employees and students at the .05-level

^2 ^One-way ANOVA. Scheffe's post-hoc tests: Business economics students statistically different from nursing students at the .05-level

In Table 2 significant group differences are investigated further with multivariate statistics, in order to identify factors independently correlated with the mean ATP scores.

**Table 2:** Pearson correlation between Attitudes Toward Prisoners scale (ATP) sum scores and demographic variables in the total study sample and the sub-samples, and results from the ensuing linear regression analyses, entering all variables found to correlate significantly with the ATP scores

		**Regression analysis with ATP score as dependent variable**			
		
**Sample**	**Pearson's correlation between ATP and other variables **(p-value, 2-tailed)	Variable entered by the forward conditional method	Variable retained in final model	Coefficients: Standardized β and p-value	Model summary: R square
Independent variable					
**Total study sample**					
Age	.12 (.001)	*	*	.15 <.001	
Gender	-.03 (.45)				
Inmates vs. residual groups	-.35 (<.001)	*	*	-.36 <.001	.14

**Prison inmates**					
Age	-.001 (.98)				
Gender	.04 (.52)				
Remanded vs. convicted	.12 (.05)	-	-	-	-
Number of previous convictions	.03 (.58)				

**Prison employees**					
Age	.22 (<.001)	*	*	.12 .04	
Gender	.13 (.01)	*			
Years of work experience	.06 (.23)				
Working in male vs. female prison	.19 (<.001)	*	*	.18 <.001	
Prison officers vs. residual employees	.24 (<.001)	*	*	.19 <.001	.11

**College students**					
Age	.21 (.004)	*			
Gender	.19 (.01)	*			
Business economics vs. other subjects studied	.29 (<.001)	*	*	.29 <.001	.09

**Table 3:** Prison inmates', prison employees' and college students' answers on questions aimed at gauging assumptions and opinions regarding prisoners, crime and punishment.

	**PRISON INMATES N = 305**			**PRISON EMPLOYEES N = 390**			**COLLEGE STUDENTS N = 184**		
**QUESTION**	**Mean (SD)**	**Median**	**Correlation with ATP**	**Mean (SD)**	**Median**	**Correlation with ATP**	**Mean (SD)**	**Median**	**Correlation with ATP**

The percentage of all crimes that are detected and punished	41 (24.3)	40	-.03	42 (23.4)	40	.00	43 (20.6)	50	.15
The percentage of all men that have ever been to prison	26^2 ^(23.4)	20	-.22^1^	7^2 ^(9.8)	5	-.09	11^2 ^(11.2)	7	-.02
The percentage of all women that have ever been to prison	13^2 ^(17.1)	6	-.20^1^	3^2 ^(6.4)	1	-.07	6^2 ^(7.1)	3	-.01
The percentage of prisoners having drug problems or alcohol problems	63^2 ^(25.3)	70	-.08	68^2 ^(16.9)	70	-.17^1^	56^2 ^(20.9)	60	.00
The percentage of prisoners having mental problems	47 (26.8)	50	-.04	49 (22.3)	50	-.01	54^2 ^(23.6)	50	.00
The percentage of prisoners that are serving time for a violent crime	30 (18.7)	27	-.16^1^	28 (14.9)	25	-.18^1^	34^2 ^(16.7)	30	.04
The percentage of all prisoners that are serving time for a sex offence	16 (15.8)	10	-.20^1^	9^2 ^(7.3)	6	-.20^1^	16 (12.0)	10	-.09
How many of those serving their first sentence will be back in prison sooner or later	58 (27.9)	60	-.13	60 (19.9)	65	-.14^1^	55 (20.6)	60	-.10

Significant group differences and correlations with ATP are marked.

^1 ^Correlation is significant at the .01 level (2-tailed)

^2 ^One-way ANOVA with Scheffe's post-hoc test: the group is significantly different from the other groups at the .05 level

**Table 4:** Prison inmates', prison employees' and college students' answers on questions aimed at gauging assumptions and opinions regarding prisoners, crime and punishment.

	**PRISON INMATES N = 305**				**PRISON EMPLOYEES N = 390**				**COLLEGE STUDENTS N = 184**				**Pearson's Chi Square**	
**QUESTION**	**Yes**	**No**	**Don't know**	**Correlation with ATP^1^**	**Yes**	**No**	**Don't know**	**Correlation with ATP^1^**	**Yes**	**No**	**Don't know**	**Correlation with ATP^1^**	**Value (DF)**	**p-value**

Do you think crime is on the rise in Norway? (%)	77	9	14	.11	84	10	7	.19^2^	88	3	8	.06	19.2 (4)	.001
Does the prison system function as it should towards the inmates? (%)	19	66	15	.14	13	73	14	-.08	87	5	8	.20^2^	353.0 (4)	<.001
Would it be better if all prisons were closed down? (%)	18	67	16	-.11	5	91	5	-.25^2^	1	95	5	-.04	89.6 (4)	<.001
	Too severe	About right	Too mild		Too severe	About right	Too mild		Too severe	About right	Too mild			
How do you consider the country's crime punishment level? (%)	59	34	8	-.06	4	44	51	-.55^2^	0	23	77	-.28^2^	415.4 (4)	<.001

Results shown as distribution (%) between the answers offered. Significant group differences and correlations with ATP are marked.

^1 ^"Don't know" answers not included in the correlation analyses

^2 ^Correlation is significant at the .01 level (2-tailed).

### The questionnaire

Tables 3 and 4 present the answers from the three groups of responders. Statistically significant group differences are noted, together with the correlation between ATP scores and the different items in the questionnaire.

### Psychometric properties of the ATP

Prior to subjecting the 36 items of the ATP to PCA the suitability of the data for factor analysis was assessed. Inspection of the correlation matrix revealed the presence of a number of coefficients of .3 and above. The Kaiser-Meyer-Oklin value was .94 and the Barlett's test of sphericity reached statistical significance, both findings supporting the factorability of the correlation matrix.

PCA revealed the presence of 7 components with eigenvalues exceeding 1 and together explaining 48.9% of the variance. However, an inspection of the screeplot revealed a clear break after the first component. This one component explained 25.3% of the variance (eigenvalue 9.09) while the remaining 6 factors (eigenvalues 2.07 to 1.02) all contributed substantially less. All 36 items loaded significantly on the first component (>.3) and as many as 20 items had loadings >.5. None of the other 6 components had item loadings >.5. For details, see Additional files 1 and 2.

In our study the scale's Cronbach alpha coefficients were .88, .91 and .93, respectively, in the three sub-samples analysed.

## Discussion

### The ATP

The results confirmed our hypotheses about significant group differences in the attitudes towards prisoners, as held by prisoners, prison employees and college students. While ATP in the whole sample differed significantly according to age, gender and group, in multivariate analysis only group remained significantly correlated with ATP, when controlling for age and gender.

As expected, the most positive attitudes were held by the prison inmates themselves. If not of an unrealistic nature, such positive attitudes should be able to enhance the effectiveness of the various relapse prevention programs offered in the correctional setting. It might, however, be problematic if those intended to help the inmates in the rehabilitation process, i.e. the prison officers, hold substantially different attitudes than the prisoners themselves do.

Remanded prisoners held less positive attitudes than convicted prisoners. Maybe remanded prisoners identify less with prisoners in general and accordingly hold attitudes more equivalent to those found in the general population.

Among prison employees, a couple of important associations emerged and were retained in the subsequent multivariate analyses. Firstly, attitudes varied significantly relative to the gender of the inmates, so that employees in female-only prisons held more positive attitudes than those employed in male-only prisons. As the female prisons were smaller than the male prisons, we can not dismiss the possibility that the more positive attitudes held by employees in the female prisons at least partly were due to prison size. The differences are contrary to Murphy and Brown who in their general population study from 2000 [15] found no significant difference in attitudes towards male and female offenders, as measured by the ATP.

The prison officers' attitudes were fairly negative and comparable to the attitudes held by a number of college students. Within the group of college students, however, those studying business economics held significantly more negative attitudes than those studying nursing. Thus, students that had chosen a "caring" profession held the most positive attitudes. Again, our findings are in disagreement with the findings of Murphy and Brown [15]. They compared mean ATP scores in people with "female", "masculine" and "neutral" occupations and found no significant differences in attitudes.

Although the original ATP scale was developed in another country, the USA, and more than 20 years ago [2], those results and ours are remarkably similar. In their original work Melvin et al found a mean ATP score of 109.5 among prisoners; we found 106. They found a mean ATP score of 90.7 for correctional officers; we found 90. The original work had included a group of people working in various prison reform and rehabilitation settings; on average, these respondents scored 108.3 on the ATP. Our more heterogeneous group of prison employees scored somewhat lower, on average 98, but even so significantly higher than the prison officers. Melvin et al's student sample averaged 90.5 on the ATP; ours averaged 91. The close correspondence between the two studies lends support to the validity of the ATP.

The ATP has also been used in countries outside the USA. Hogue [10] used it to assess the attitudes held by correctional staff in Great Britain. Although the relative ranking of the mean ATP scores in the different groups investigated were similar to ours, the British scores were overall somewhat lower. A Spanish study [12] demonstrated substantially lower scores in correctional officers than in prison rehabilitation team members. The same Spanish study found students' mean ATP scores similar to those found in our student sample, while a British population study [15] found somewhat lower mean ATP scores.

The above studies have all used the ATP. However, a number of studies using other instruments than the ATP merit attention. These studies have demonstrated that older prison officers seem to focus more on the rehabilitative aspects of their work [7,14,22] and hold more optimistic attitudes towards the inmates than younger officers do [8]. The findings regarding the effect of the prison officers' gender on attitudes have been inconsistent; with either no effect [8,9] or female officers holding more positive attitudes [7]. In the multivariate analyses carried out in our study, age and gender did not contribute significantly. While other studies have reported inconsistent results regarding the effect of the amount of previous correctional work experience [4,6-9,11], we did not find any significant effect of work experience on the mean ATP score.

There is a paucity of studies reporting on the attitudes of correctional personnel in administrative positions [13]. It seems to be fairly unique that our study is able to report on the attitudes held by prison officers and other employees in the same prison facilities. As already stated, we found that prison officers held more negative attitudes than employees in other work positions. It might be that working with prisoners on a day-to-day basis leads to more negative attitudes. As the main task of the prison officers is to supervise and control the inmates and their behaviour, they must to a certain degree focus on negative aspects of the individual. Other prison employees, particularly teachers and people running rehabilitation programs, are expected to focus more on the inmates' resources and potentials. In addition, they have at their disposal programs and treatments that may provide a sense of empowerment and optimism. Together, these circumstances may result in more positive attitudes.

It is important to acknowledge that attitudes do not necessarily translate into action [18]. Furthermore, some of the attitudes conceived as negative according to the ATP may in fact represent a fair evaluation of some of the prisoners. Some offenders may be better served by a realistic assessment of their problems rather than by unjustified optimism as to their capacity for change. Hence, both extremely low and extremely high ATP scores may be counterproductive, at least in a forensic setting. Even so, the extremely wide gaps between the highest and the lowest ATP scores within all groups investigated (Table 1) are noteworthy. Intuitively, it seems more reasonable that students would present a wider range of scores than prison officers who have had more uniform experiences with prisoners through their work.

### The questionnaire

As shown in Tables 3 and 4, the three groups investigated differed significantly in their answers to a number of the questions. Even so, some questions received remarkably similar answers, like the ones about the percentage of crimes that are punished and the high expected re-imprisonment rate. The latter may testify to a general distrust in rehabilitation programs across all groups. The prisoners gave the highest estimates about the number of the general population that has been incarcerated. This may partly be explained by imprisonment being more prevalent in their home environment.

All, including the prisoners themselves perceived prisoners as a psychologically deviant group with frequent drug and alcohol abuse and other mental problems. These beliefs have implications for the attitudes held towards prisoners, particularly among prison officers [16].

Both prisoners and students estimated the proportion of prisoners that had committed sexual offences to be higher than the prison officers did, with the prison officers' estimates being closer to the national average of about 7% [23]. The groups' mean estimate of the percentage of prisoners having committed a violent crime, 28–34%, is slightly above the national average of about 25% [23].

Not surprising, most inmates were of the opinion that the crime punishment level in Norway is too severe. However, as many as 1 in 3 found it about right and some even too mild. Most prison employees found the punishment level adequate or too mild, while the students expressed the most punitive opinion: more than 3 in 4 found the punishment level too mild. Unsolicited, a fair number of respondents from all groups had indicated in the margin of the questionnaire that they found the punishment level to be too severe in cases of drug related crimes and too mild in relation to sex offences. This may indicate that attitudes towards prisoners most likely vary, according to the crimes they have been convicted of.

Although prison inmates and prison employees, both of them groups that hold first-hand knowledge about the issue, agreed that our prisons do not function as they should, only a small minority in both groups believed that we would be better off if all prisons were shut down. The vast majority of students expressed satisfaction with the way our prisons function. This may be taken to indicate that, as long as our prisons keep offenders locked up, most people are of the opinion that our prisons function according to intention.

A number of the answers to the questionnaire (Tables 3 and 4) correlated strongly with the ATP, particularly in prison officers, and invariably in such a way that those who expressed more pessimistic and punitive views had a lower ATP score. This finding testifies to the close relationship between a person's attitudes towards prisoners and the same person's general beliefs regarding prisoners and crime and punishment in society.

### The psychometric properties of ATP

To our knowledge, this is the first time the scale has been subjected to a PCA. The results of the factor analysis support the claim that the ATP taps one basic construct. At the same time, the results suggest that the number of items in ATP, 36, could be drastically reduced without losing the psychometric properties of the scale. Such an item reduction would make the instrument more user-friendly.

### Limitations of the study

In assessing the results of the present study, a number of limitations must be taken into account.

A comprehensive study of attitudes towards prisoners should adopt a broader, more diversified approach. In particular, more than just one instrument should be used.

As always in this kind of studies, one is confronted with the question about statistically vs. clinically significant findings. We have no way of stipulating what would constitute clinically significant group differences in the ATP scores; all we can do is caution against making too strong inferences regarding the results presented.

While attrition was very low among college students, it was higher in the prisons, particularly among inmates in one of them. Unfortunately, we had no opportunity to conduct proper analyses of attrition. Some prisoners were on leave, either for social reasons or because they had to appear in court. In addition, we have missed prisoners with mental or somatic conditions rendering them unable to participate and all prisoners that were not able to answer in English or Norwegian. Hence, the sample is not representative for those groups of inmates.

The differences in attrition rates between prisons could be problematic if there was a link between negative attitudes and high attrition rates, with employees in some prisons being of the opinion that it is a waste of time to do research on prisoners. We do not have any indication that this was the case in our study.

Correctional institutions may differ in a number of ways: some are well run, some are chaotic; some have a stronger focus on rehabilitation, others on punishment. Such factors may influence attrition rates and the attitudes held by employees and inmates. These issues have not been addressed by us but should be explored in future studies.

Although ATP scores for a group of college students may be representative for young people, they should not be taken to indicate the attitudes held towards prisoners in the population at large. Ideally, respondents should be drawn at random from the total population and be large enough to allow the exploration of how gender, age, socio-economic status, crime victimization and education influence people's attitudes towards offenders. However, a group of college students is an interesting comparison group to prison inmates. They may in some respects be considered complementary groups: as opposed to young prisoners, most students have been successfully integrated into a society from which the prisoners have been forcefully excluded, but will have to re-enter after serving their sentence. On the other hand, one would expect college students and prisoners to have markedly different socio-economic backgrounds. This could be an important confounder.

Lastly, the ATP was developed for use in the USA. Even though our results are remarkably similar to those of Melvin et al's original work, we have to take into account that there might be differences in culture and crime policy between the USA and Norway that makes the scale less suitable to measure what it originally intended.

## Conclusion

In society at large the demand for long prison sentences and punitive measures towards prisoners, particularly violent perpetrators and sexual offenders, is strong. Most prisons offer various rehabilitation programs intended to reduce the likelihood of re-offending after release. In this active setting, prison officers and other prison employees are engaged in order to assist in the process of change. Prison employees holding positive attitudes towards the inmates have been shown to significantly ameliorate tension, strain and conflict in the prison community [8]. All prisoners will eventually be released back into the community. In this context, the attitudes held toward prisoners, both among prison employees and in the general public, become important [24]. Future research should try to investigate further how prison employees' attitudes influence the effectiveness of the various rehabilitation programs offered in our prisons and to what extent such attitudes may be influenced by educational programs and the dispersion of factual information.

## Competing interests

The authors declare that they have no competing interests.

## Authors' contributions

E. Kjelsberg and A.-B. Rustad developed the research protocol and the questionnaire. T. H. Skoglund and A.-B. Rustad were responsible for data collection at the participating sites. T. H. Skoglund undertook a comprehensive literature review. E. Kjelsberg performed the statistical analyses and drafted the first version of the manuscript. All authors read, gave feedback, and approved the final manuscript.

## Pre-publication history

The pre-publication history for this paper can be accessed here:



## Supplementary Material

Additional data file 1 Principal components analysis of the 36 items in the Attitudes Toward Prisoners scale: Total variance explained. Click here for file

Additional data file 2 Principal components analysis of the 36 items in the Attitudes Toward Prisoners scale: Component matrix. â€™râ€™ signifies that the scoring of the item has been reversed. Click here for file
