# Supplementary material for: Attitudes towards prisoners, as reported by prison inmates, prison employees and college students
Source: BMC Public Health. 2007 May 4;7:71. doi: 10.1186/1471-2458-7-71 (PMC1891097; doi:10.1186/1471-2458-7-71)
Supplement: Additional data file 1 — Principal components analysis of the 36 items in the Attitudes Toward Prisoners scale: Total variance explained. [file 1471-2458-7-71-S1.doc]

Additional file 1. Principal components analysis of the 36 items in the Attitudes Toward Prisoners scale: Total variance explained

| Component | Initial Eigenvalues | | | Extraction Sums of Squared Loadings | | |
| --- | --- | --- | --- | --- | --- | --- |
|  | Total | % of Variance | Cumulative % | Total | % of Variance | Cumulative % |
| 1 | 9,093 | 25,257 | 25,257 | 9,093 | 25,257 | 25,257 |
| 2 | 2,067 | 5,742 | 30,999 | 2,067 | 5,742 | 30,999 |
| 3 | 1,812 | 5,032 | 36,031 | 1,812 | 5,032 | 36,031 |
| 4 | 1,363 | 3,786 | 39,817 | 1,363 | 3,786 | 39,817 |
| 5 | 1,155 | 3,208 | 43,025 | 1,155 | 3,208 | 43,025 |
| 6 | 1,106 | 3,072 | 46,097 | 1,106 | 3,072 | 46,097 |
| 7 | 1,021 | 2,835 | 48,932 | 1,021 | 2,835 | 48,932 |
| 8 | ,987 | 2,741 | 51,673 |  |  |  |
| 9 | ,926 | 2,571 | 54,244 |  |  |  |
| 10 | ,877 | 2,436 | 56,680 |  |  |  |
| 11 | ,863 | 2,397 | 59,077 |  |  |  |
| 12 | ,828 | 2,301 | 61,378 |  |  |  |
| 13 | ,818 | 2,273 | 63,651 |  |  |  |
| 14 | ,784 | 2,178 | 65,829 |  |  |  |
| 15 | ,769 | 2,137 | 67,966 |  |  |  |
| 16 | ,755 | 2,097 | 70,063 |  |  |  |
| 17 | ,724 | 2,011 | 72,074 |  |  |  |
| 18 | ,685 | 1,903 | 73,976 |  |  |  |
| 19 | ,671 | 1,865 | 75,842 |  |  |  |
| 20 | ,655 | 1,818 | 77,660 |  |  |  |
| 21 | ,623 | 1,732 | 79,391 |  |  |  |
| 22 | ,609 | 1,693 | 81,084 |  |  |  |
| 23 | ,586 | 1,628 | 82,712 |  |  |  |
| 24 | ,584 | 1,622 | 84,334 |  |  |  |
| 25 | ,562 | 1,562 | 85,896 |  |  |  |
| 26 | ,537 | 1,491 | 87,387 |  |  |  |
| 27 | ,531 | 1,474 | 88,861 |  |  |  |
| 28 | ,507 | 1,410 | 90,270 |  |  |  |
| 29 | ,486 | 1,350 | 91,620 |  |  |  |
| 30 | ,481 | 1,337 | 92,958 |  |  |  |
| 31 | ,459 | 1,275 | 94,233 |  |  |  |
| 32 | ,453 | 1,258 | 95,490 |  |  |  |
| 33 | ,437 | 1,215 | 96,705 |  |  |  |
| 34 | ,421 | 1,169 | 97,874 |  |  |  |
| 35 | ,405 | 1,124 | 98,998 |  |  |  |
| 36 | ,361 | 1,002 | 100,000 |  |  |  |

Extraction Method: Principal Component Analysis.
