# Supplementary material for: Attitudes towards prisoners, as reported by prison inmates, prison employees and college students
Source: BMC Public Health. 2007 May 4;7:71. doi: 10.1186/1471-2458-7-71 (PMC1891097; doi:10.1186/1471-2458-7-71)
Supplement: Additional data file 2 — Principal components analysis of the 36 items in the Attitudes Toward Prisoners scale: Component matrix. â€™râ€™ signifies that the scoring of the item has been reversed. [file 1471-2458-7-71-S2.doc]

Additional file 2. Principal components analysis of the 36 items in the Attitudes Toward Prisoners scale: Component matrix. ’r’ signifies that the scoring of the item has been reversed.

|  | Component | | | | | | |
| --- | --- | --- | --- | --- | --- | --- | --- |
|  | 1 | 2 | 3 | 4 | 5 | 6 | 7 |
| ratp19 | ,657 |  |  |  |  |  |  |
| ratp25 | ,639 | -,327 |  |  |  |  |  |
| ratp29 | ,628 |  |  |  |  |  |  |
| ratp31 | ,613 |  |  |  |  |  |  |
| ratp30 | ,612 |  |  |  |  |  | -,320 |
| ratp9 | ,580 |  | -,355 |  |  |  |  |
| atp26 | ,566 |  |  |  |  |  |  |
| atp21 | ,558 |  |  |  |  |  |  |
| atp23 | ,557 |  |  | -,323 |  |  |  |
| ratp27 | ,552 |  |  | -,318 |  |  |  |
| ratp16 | ,543 |  | -,375 |  |  |  |  |
| ratp14 | ,530 |  | -,324 |  |  |  |  |
| ratp35 | ,527 | -,308 |  |  |  |  |  |
| ratp22 | ,526 | -,391 |  |  |  |  |  |
| atp32 | ,525 |  |  | ,335 |  | -,356 |  |
| ratp12 | ,524 |  |  |  |  |  |  |
| ratp24 | ,517 | -,311 | ,307 |  |  |  |  |
| atp28 | ,515 |  |  |  | -,335 |  |  |
| atp34 | ,513 | ,444 |  |  |  |  |  |
| ratp6 | ,506 |  | -,468 |  |  |  |  |
| ratp1 | ,491 |  |  |  |  |  |  |
| atp33 | ,485 |  |  |  | -,409 |  |  |
| atp36 | ,479 | ,424 |  |  |  |  |  |
| atp18 | ,479 |  |  |  |  |  |  |
| atp15 | ,477 |  |  |  |  |  |  |
| atp7 | ,457 |  | ,304 |  |  |  |  |
| ratp13 | ,438 |  |  | ,340 |  |  |  |
| atp20 | ,429 | ,348 |  |  |  |  |  |
| ratp3 | ,423 |  |  |  |  |  |  |
| ratp10 | ,417 | -,399 |  |  |  |  |  |
| atp11 | ,369 |  |  |  |  | ,349 |  |
| ratp17 | ,358 | -,410 |  |  |  | ,334 |  |
| atp5 | ,321 |  | ,392 | ,307 |  |  |  |
| atp4 | ,374 |  |  | ,414 |  |  |  |
| atp8 | ,305 |  | ,316 |  | ,389 |  |  |
| atp2 | ,303 |  |  |  |  | ,316 | ,383 |

Extraction Method: Principal Component Analysis.
